# *Molecular Psychiatry*, August 2020: new impact factor, and highlights of recent advances in psychiatry, including an overview of the brain’s response to stress during infection with the severe acute respiratory syndrome coronavirus 2 (SARS-CoV-2)

**DOI:** 10.1038/s41380-020-0845-y

**Published:** 2020-07-28

**Authors:** Julio Licinio, Ma-Li Wong

**Affiliations:** State University of New York, Upstate Medical University, Syracuse, NY 13210 USA

**Keywords:** Schizophrenia, ADHD

We are delighted that the 2019 Impact Factor (IF) figures have recently been released by the Journal Citation Reports® Science Edition (Clarivate Analytics, 2020). As you know, the IF is calculated as total citations in 2019 of all papers published by a journal in 2017 + 2018 divided by the total numbers of full articles published in 2017 + 2018. I am delighted that the 2019 Impact Factor for *Molecular Psychiatry* is now 12.384 (an increase from 11.973 in the previous year). What is particularly remarkable is that we have achieved that while simultaneously increasing the number of outstanding work that we publish.

*Molecular Psychiatry*’s 2019 Journal Citation Reports ranking is as follows: 6th of 155 in Psychiatry, 10th of 271 in Neuroscience, and 11th of 297 in Biochemistry & Molecular Biology. We would like to sincerely thank all of you, authors, readers, and editorial board members, for your support of *Molecular Psychiatry* over the years, which has been essential for the journal to achieve its high level of continued success.

This year we published four special issues, one on schizophrenia [1–19], one on stress and behavior [20–38], and two on depression, in June [39–55] and July [56–76]. We now go back to our regular publishing schedule, highlighting in this issue incredible progress in several different areas of psychiatry. This editorial will highlight some of that progress.

This exciting issue starts with a guest editorial by Steenblock et al., on the impact of SARS-CoV-2 infection and the neuroendocrine stress axis [77]. We then have two interesting Perspectives, one by the Consortium on Vulnerability to Externalizing Disorders and Addictions (c-VEDA), in which they examine an accelerated longitudinal cohort of children and adolescents in India [78], and the other by Thapar & Riglin commenting on the importance of a developmental perspective in Psychiatry, and answering the essential question: what do recent genetic-epidemiological findings show? [79] These exciting Perspectives are followed by three important Reviews: by Prieto et al. on post-translational modifications of the Fragile X Mental Retardation Protein in neuronal function and dysfunction; by Rogdaki et al., a meta-analysis of the magnitude and heterogeneity of brain structural abnormalities in 22q11.2 deletion syndrome [80], and by Smigielski et al., a systematic review of empirical human findings on the epigenetic mechanisms in schizophrenia and other psychotic disorders [81].

We have Immediate Communications as a unique manuscript type in *Molecular Psychiatry*. Those papers are rapidly reviewed, and accepted only if the reviews are particularly favorable. They are then prioritized for publication. We have three Immediate Communications in this issue. Dempster et al. present a 7-T magnetic resonance spectroscopic study of glutathione and glutamate showing early treatment response in first-episode psychosis [82]; Niculescu et al. present intriguing work on blood biomarkers for memory that can be targeted toward early detection of risk for Alzheimer’s disease (AD), pharmacogenomics, and repurposed drugs [83], and Polimanti et al. leverage genome-wide data to investigate differences between opioid use versus opioid dependence in 41,176 individuals from the Psychiatric Genomics Consortium [84].

Regular articles in this issue start with the paper that is the topic of our Image Section, in which we highlight striking figures that illustrate important new discoveries. In that article, Du et al. show that cocaine-induced ischemia in the prefrontal cortex is associated with escalation of cocaine intake in rodents [85]. In other original research articles, we present key new advances across multiple fields. Kendler et al. show that the risk for drug abuse, alcohol use disorder, and psychosocial dysfunction in offspring from high-density pedigrees is moderated by personal, family, and community factors [86]. Harrison et al. present a systematic review and meta-analysis of the neuropathology of bipolar disorder [87]. Brikell et al. examine the contribution of common genetic risk variants for attention-deficit/hyperactivity disorder (ADHD) to a general factor of childhood psychopathology. Their results suggest that common genetic risk variants associated with ADHD, and captured by polygenic risk scores (PRS), also influence a general genetic liability towards broad childhood psychopathology in the general population, in addition to a specific association with hyperactivity/impulsivity symptoms [88].

In our beautiful cover article, Sun et al. conducted large-scale mapping of cortical alterations in 22q11.2 deletion syndrome demonstrating convergence with idiopathic psychosis and effects of deletion size [89]. They found a robust neuroanatomic signature of 22q11DS, and the first evidence that deletion size impacts brain structure and that psychotic illness in this highly penetrant deletion was associated with similar neuroanatomic abnormalities to idiopathic schizophrenia.

Our original research articles continue with other remarkable papers. Amal et al. provide evidence that mutation in a mouse model of autism leads to changes in the S-nitroso-proteome and affects key proteins involved in vesicle release and synaptic function [90]. In a randomized clinical trial conducted in Japan, Yamasue et al. studied the effects of intranasal oxytocin on the core social symptoms of autism spectrum disorder (ADS) [91]. They did not find highly beneficial clinical outcomes other than on repetitive behavior and gaze fixation on socially relevant regions. There were no significant differences in the prevalence of adverse events between groups. Based on their findings, they cannot recommend continuous intranasal oxytocin treatment alone at the current dose and duration for treatment of the core social symptoms of high-functioning ASD in adult men, although this large-scale trial suggests oxytocin’s possibility to treat ASD repetitive behavior. Bis et al. used whole-exome sequencing to identify novel rare and common AD-associated variants involved in immune response and transcriptional regulation [92]. In our last original article, Temido-Ferreira shows that age-related shift in long-term depression (LTD), is dependent on neuronal adenosine A2A receptors (A2AR) interplay with metabotropic glutamate receptor type 5 (mGluR5) and N-Methyl-d-aspartate (NMDA) receptors [93]. The authors suggest that this A2AR/mGluR5/NMDAR interaction might prove a suitable alternative for regulating aberrant mGluR5/NMDAR signaling in AD without disrupting their constitutive activity.

These superb articles across a variety of field of great importance to the broad field of molecular psychiatry demonstrate that *Molecular Psychiatry*, with a new IF of 12.384, is now the premier journal in our field that integrates clinical psychiatry with molecular neurobiology, genetics/genomics, imaging, and other state-of-the-art approaches that unravel the pathways and mechanisms underlying psychiatric disorders.
